# Loss of MTAP expression is strongly linked to homozygous 9p21 deletion, unfavorable tumor phenotype, and noninflamed microenvironment in urothelial bladder cancer

**DOI:** 10.1002/2056-4538.70012

**Published:** 2024-12-12

**Authors:** Natalia Gorbokon, Niklas Wößner, Viktoria Ahlburg, Henning Plage, Sebastian Hofbauer, Kira Furlano, Sarah Weinberger, Paul Giacomo Bruch, Simon Schallenberg, Florian Roßner, Sefer Elezkurtaj, Maximilian Lennartz, Niclas C Blessin, Andreas H Marx, Henrik Samtleben, Margit Fisch, Michael Rink, Marcin Slojewski, Krystian Kaczmarek, Thorsten Ecke, Tobias Klatte, Stefan Koch, Nico Adamini, Sarah Minner, Ronald Simon, Guido Sauter, Henrik Zecha, David Horst, Thorsten Schlomm, Lukas Bubendorf, Martina Kluth

**Affiliations:** ^1^ Institute of Pathology University Medical Center Hamburg‐Eppendorf Hamburg Germany; ^2^ Department of Urology Charité Berlin Berlin Germany; ^3^ Institute of Pathology Charité Berlin Berlin Germany; ^4^ Department of Pathology Academic Hospital Fuerth Fuerth Germany; ^5^ Department of Urology University Medical Center Hamburg‐Eppendorf Hamburg Germany; ^6^ Department of Urology Marienhospital Hamburg Hamburg Germany; ^7^ Department of Urology and Urological Oncology Pomeranian Medical University Szczecin Poland; ^8^ Department of Urology Helios Hospital Bad Saarow Bad Saarow Germany; ^9^ Department of Pathology Helios Hospital Bad Saarow Bad Saarow Germany; ^10^ Department of Urology Albertinen Hospital Hamburg Germany; ^11^ Institute of Pathology University Hospital Basel Basel Switzerland

**Keywords:** MTAP, 9p21 deletion, tissue microarray, FISH, immunohistochemistry, urothelial bladder carcinoma

## Abstract

Homozygous 9p21 deletions usually result in a complete loss of S‐methyl‐5′‐thioadenosine phosphorylase (MTAP) expression visualizable by immunohistochemistry (IHC). MTAP deficiency has been proposed as a marker for predicting targeted treatment response. A tissue microarray including 2,710 urothelial bladder carcinomas were analyzed for 9p21 deletion by fluorescence *in situ* hybridization and MTAP expression by IHC. Data were compared with data on tumor phenotype, patient survival, intratumoral lymphocyte subsets, and PD‐L1 expression. The 9p21 deletion rate increased from pTaG2 low (9.2% homozygous, 25.8% heterozygous) to pTaG2 high (32.6%, 20.9%; *p* < 0.0001) but was slightly lower in pTaG3 (16.7%, 16.7%) tumors. In pT2–4 carcinomas, 23.3% homozygous and 17.9% heterozygous deletions were found, and deletions were tied to advanced pT (*p* = 0.0014) and poor overall survival (*p* = 0.0461). Complete MTAP loss was seen in 98.4% of homozygous deleted while only 1.6% of MTAP negative tumors had retained 9p21 copies (*p* < 0.0001). MTAP loss was linked to advanced stage and poor overall survival in pT2–4 carcinomas (*p* < 0.05 each). The relationship between 9p21 deletions/MTAP loss and poor patient prognosis was independent of pT and pN (*p* < 0.05 each). The 9p21 deletions were associated with a noninflamed microenvironment (*p* < 0.05). Complete MTAP loss is strongly tied to homozygous 9p21 deletion, aggressive disease, and noninflamed microenvironment. Drugs targeting MTAP‐deficiency may be useful in urothelial bladder carcinoma. MTAP IHC is a near perfect surrogate for MTAP deficiency in this tumor type.

## Introduction

The enzyme S‐methyl‐5′‐thioadenosine phosphorylase (MTAP) is of topical interest as an indirect drug target as well as a predictive and diagnostic marker in urothelial carcinoma. MTAP is critical to the production of adenine which is a structural component of both DNA and RNA and thus needed for cell replication. Adenine can only be built either by MTAP independent *de novo* synthesis or by recycling from adenine containing molecules through the MTAP dependent salvage pathway [1]. The *MTAP* gene is located at 9p21.3, only 30 kb apart from the *cyclin dependent kinase inhibitor 2A* (*CDKN2A*) gene which is homozygous deleted in up to 15% of all human cancers [2, 3, 4]. Homozygous co‐deletions of *MTAP* occur in 80–90% of *CDKN2A* deleted tumor cells [5]. In MTAP‐deficient tumor cells, adenine synthesis is solely dependent on *de novo* biosynthesis, which directly or indirectly involves several druggable enzymes including dihydrofolate reductase and phosphoribosylglycinamide formyltransferase [6]. MTAP‐deficient cells can also be targeted by drugs inhibiting protein arginine N‐methyltransferase 5 (PRMT5) and methionine adenosyltransferase 2A (MAT2A)  [7]. PRMT5 is an essential enzyme for methylation of numerous proteins [8] while MAT2A is essential for the synthesis of S‐adenosylmethionine, the methyl donor and substrate of PRMT5 [9, 10]. PRMT5 is upregulated and involved in the progression of various cancers [7]. Because PRMT5 is partially inhibited by the intracellular accumulation of the unprocessed MTAP metabolite 5′‐deoxy‐5′‐methylthioadenosine (MTA) [11], MTAP deficiency makes cells vulnerable to drugs targeting PRMT5 or MAT2A [7]. A clinical phase 1 trial has shown significant size reduction of *MTAP*‐deleted cancers after treatment with the PRMT5 inhibitor MRTX1719 [12]. Other clinical trials employing drugs targeting PRMT5 are ongoing [13]. Also of interest, both MTAP deficiency [14] and *CDKN2A* loss [15] could be linked to poor response to immune checkpoint inhibitors (CPIs) in urothelial carcinoma. The 9p21 loss was also associated with poor response to CPIs in lung cancer, melanoma, and miscellaneous solid tumors [16].

MTAP is ubiquitously expressed in normal cells, and MTAP deficiency can be detected by immunohistochemistry (IHC). Studies in malignant mesothelioma, a cancer that often shows homozygous 9p21 deletions, have shown that MTAP IHC can serve as a diagnostic tool for the distinction of malignant from benign mesothelial proliferations [17, 18]. Urothelial carcinoma is another tumor entity with a high rate of 9p21 deletions, which could be treated by drugs targeting MTAP deficiency [19]. Homozygous 9p21 deletions have been described in 5–83% of urothelial carcinomas [20, 21, 22, 23]. Only one recent study analyzed MTAP expression by IHC and found MTAP loss in 22% of 729 consecutive urothelial cancer cases [24]. Considering that urothelial carcinomas are often treated by CPIs and that considerable diagnostic difficulties exist in the diagnosis of low‐grade neoplasms/flat dysplasia [25] and in urine cytology [26], immunohistochemical MTAP analysis could also be of diagnostic and predictive value in this tumor type.

In this study, we aimed to understand the prevalence of MTAP deficiency in urothelial carcinoma, and to determine its relationship with parameters of the immune microenvironment, clinicopathological parameters, and disease outcome. Over 2,700 urothelial carcinomas were analyzed in a tissue microarray (TMA) by fluorescence *in situ* hybridization (FISH) and IHC.

## Materials and methods

### Tissue microarrays

A set of 10 TMA blocks containing between 89 and 694 urothelial bladder carcinomas each (a total of 2,971 bladder cancers with one punch per tumor each) was used for this study. Nine of these TMA blocks with 2,710 tumors were used for 9p copy number analysis, and eight TMA blocks with 2,466 tumors were used for MTAP expression analysis. Matched analysis of both 9p copy numbers and MTAP expression was performed on seven TMA blocks (with 2,205 tumors). All tumors were retrieved from the archives of the Institute of Pathology, University Hospital Hamburg, Germany, Institute of Pathology, Charité Berlin, Germany, Department of Pathology, Academic Hospital Fuerth, Germany, or Department of Pathology, Helios Hospital Bad Saarow, Germany, and/or treated at Department of Urology, University Hospital Hamburg, Germany, Department of Urology, Charité Berlin, Germany, Department of Urology, Helios Hospital Bad Saarow, Germany, Department of Urology, Albertinen Hospital, Hamburg, Germany, and Department of Urology and Urological Oncology, Pomeranian Medical University, Szczecin, Poland. Patients from each center were treated according to the guidelines at the time. In brief, patients with pTa/pT1 disease underwent a transurethral resection of the bladder tumor with or without postoperative or adjuvant instillation therapy, while most patients with pT2–4 disease were treated by radical cystectomy. Available histopathological data including grade, tumor stage (pT), lymph node status (pN), and status of blood vessel (V) and lymphatic vessel (L) infiltration are shown in Table 1. Clinical follow‐up data were available from 636 patients with pT2–4 carcinomas treated by cystectomy [overall survival (OS), 1–176 months, median 15 months]. The cohort included 512 patients with muscle‐invasive carcinomas who had never been treated with CPIs. The tissues were fixed in 4% buffered formalin and then embedded in paraffin. TMA manufacturing was as described [27, 28]. In brief, one tissue spot (diameter: 0.6 mm) per patient was transmitted from a cancer‐containing donor block into an empty recipient paraffin block. Conventional whole sections were prepared from donor tissue blocks of 10 cases each with and without immunohistochemical MTAP expression loss. The use of archived remnants of diagnostic tissues for TMA manufacturing, their analysis for research purposes, and patient data were according to local laws (HmbKHG, §12) and analysis had been approved by the local ethics committee (Ethics Commission Hamburg, WF‐049/09). All work has been carried out in compliance with the Helsinki Declaration. Data on parameters of the tumor microenvironment were available from a previous study employing the same set of the TMAs as used for MTAP IHC analysis in this study [29]. In brief, sections of the TMAs were immunostained with 21 antibodies (supplementary material, Table S1) using multiplex fluorescence IHC. Each tissue spot was analyzed with a framework of neuronal networks to identify immune cell types (CD8+ cytotoxic T cells, CD4+ T helper cells, M1 and M2 macrophages, dendritic cells) and to measure the density (cells per mm^2^) of the different immune cell types.

**Table 1:** Patient cohort

	Study cohort on TMA (*n* = 2,710)
Follow‐up	
Available data (*n*)	636
Mean (months)	26.7
Median (months)	15
Missing data (*n*)	2,074
Tumor stage, *n* (%)	
pTa	887 (39.2%)
pT2	462 (20.4%)
pT3	615 (27.2%)
pT4	298 (13.2%)
Missing data	448
Tumor grade, *n* (%)	
G2	820 (30.6%)
G3	1,858 (69.4%)
Missing data	32
Lymph node metastasis, *n* (%)	
pN0	734 (62.0%)
pN+	449 (38.0%)
Missing data	1,527
Resection margin, *n* (%)	
R0	595 (80.6%)
R1	143 (19.4%)
Missing data	1,972
Lymphatic invasion, *n* (%)	
L0	275 (49.5%)
L1	281 (50.5%)
Missing data	2,154
Venous invasion, *n* (%)	
V0	450 (74.4%)
V1	155 (25.6%)
Missing data	2,105

Percent in the column ‘study cohort on TMA’ refers to the fraction of samples with available data in each category.

### Fluorescence *in situ* hybridization

Five‐micrometer TMA sections were deparaffinized with xylol, rehydrated through a graded alcohol series, and exposed to heat‐induced denaturation for 10 min in a water bath at 99 °C in P1 pretreatment solution (Agilent Technologies, Santa Clara, CA, USA; #K5799). For proteolytic treatment, slides were added to VP2000 protease buffer (Abbott, Chicago, IL, USA; #2J.0730) for 200 min at 37 °C in a water bath. A commercial FISH probe kit containing both, a 9p21 probe including the *CDKN2A* and the *MTAP* gene and a centromere 9 probe, were utilized for 9p21 copy number detection (ZytoLight® SPEC CDKN2A/CEN 9 Dual Color Probe, Zytovision, Bremerhaven, Germany; #Z‐2063). Hybridization was performed overnight at 37 °C in a humidified chamber. Posthybridization washes were done according to the manufacturer's direction at 37 °C (Agilent Technologies; #K5799). Nuclei were counterstained with 125 ng/mL 4′,6‐diamino‐2‐phenylindole in antifade solution (Biozol, Eching, Germany; #VEC‐H‐1200). Stained sections were manually interpreted with an epifluorescence microscope and copy numbers of 9p21 and centromere 9 were estimated for each tissue spot. Presence of fewer 9p21 signals than centromere 9 probe signals in at least 60% tumor nuclei or one 9p21 and one centromere 9 signal (monosomy of chromosome 9) in nearly all tumor nuclei were considered as heterozygous deletion. Complete absence of 9p21 signals but presence of centromere 9 signals in the tumor nuclei and presence of unequivocal 9p21 and centromere 9 signals in the tumor adjacent to normal cell nuclei was considered homozygous 9p21 deletion. Tissue spots lacking any detectable 9p21 signals in all (tumor and normal cell nuclei) or lack of any normal cells as an internal control for successful hybridization of the 9p21 probe were excluded from analysis. These thresholds were based on the results of a previous study in which 100% concordance in *PTEN* copy number status was achieved using FISH and single nucleotide polymorphism array analysis in a cohort of 72 prostate cancers [30].

### Immunohistochemistry

A TMA containing urothelial bladder carcinomas with 9p21 wild type (*n* = 20), heterozygous deletions (*n* = 20), and homozygous deletion (*n* = 20) was used to titrate the antibodies to obtain maximal staining in non‐neoplastic cells while background staining was still lacking in tumors with homozygous deletion. Freshly prepared 2.5‐μm TMA sections were applied on 1 day in one experiment in a Dako Omnis automated stainer (Agilent Technologies, Santa Clara, CA, USA) using the EnVision FLEX, High pH Kit (Agilent Technologies; #GV800). Slides were deparaffinized with Clearify™ agent (Agilent Technologies; #GC810) and exposed to heat‐induced antigen retrieval for 30 min at 97 °C in Target Retrieval Solution, High pH reagent (part of Agilent kit #GV800). Primary antibody specific for MTAP (recombinant rabbit monoclonal, MSVA‐741R, MS Validated Antibodies GmbH, Hamburg, Germany; #5293‐741R) was applied at ambient temperature for 30 min at a dilution of 1:50. Endogenous peroxidase activity was blocked with Peroxidase‐Blocking‐Reagent (part of Agilent kit #GV800) for 3 min. Bound antibody was visualized using the EnVision FLEX, High pH Kit reagents DAB+ Chromogen and Substrate Buffer (parts of Agilent kit #GV800) and EnVision FLEX + Rabbit LINKER (Agilent Technologies; #GV809) according to the manufacturer's directions. The sections were counterstained with hematoxylin. For tumor tissues, the average staining intensity of unequivocally neoplastic cells was estimated as 0, 1+, 2+, and 3+. For the classification of a tumor as completely negative (0), the presence of unequivocal MTAP staining in tumor‐adjacent normal tissue was required. Tumors with complete absence of MTAP staining in tumor cells and a lack of stromal cells with unequivocal MTAP staining were considered ‘noninformative’.

### Statistics

Statistical calculations were performed with JMP17® software (SAS®, Cary, NC, USA). Contingency tables and the chi‐square test were performed to determine associations between 9p21 copy number status, MTAP immunostaining, and tumor phenotype. ANOVA analysis was used to study the relationship between MTAP deficiency and parameters of the tumor microenvironment (immune cell status). Survival curves were plotted according to the Kaplan–Meier method and the log‐rank test was applied to detect significant differences between groups. Proportional hazard analysis was used to test the independence of the impact on OS from the established prognostic parameters (pT and pN).

## Results

### Technical issues

A total of 1,792 (72.7%) of 2,466 urothelial carcinomas were interpretable for MTAP IHC and 1,711 of 2,710 (63.1%) were evaluable for 9p21 deletion assessment by FISH. Noninterpretable tumors were caused by insufficient hybridization (FISH), absence of MTAP immunostaining of both tumor cells and stroma cells (IHC), lack of unequivocal tumor cells on the TMA spots, or absence of entire tissue spots on the TMA.

### MTAP IHC

**Figure 1:**
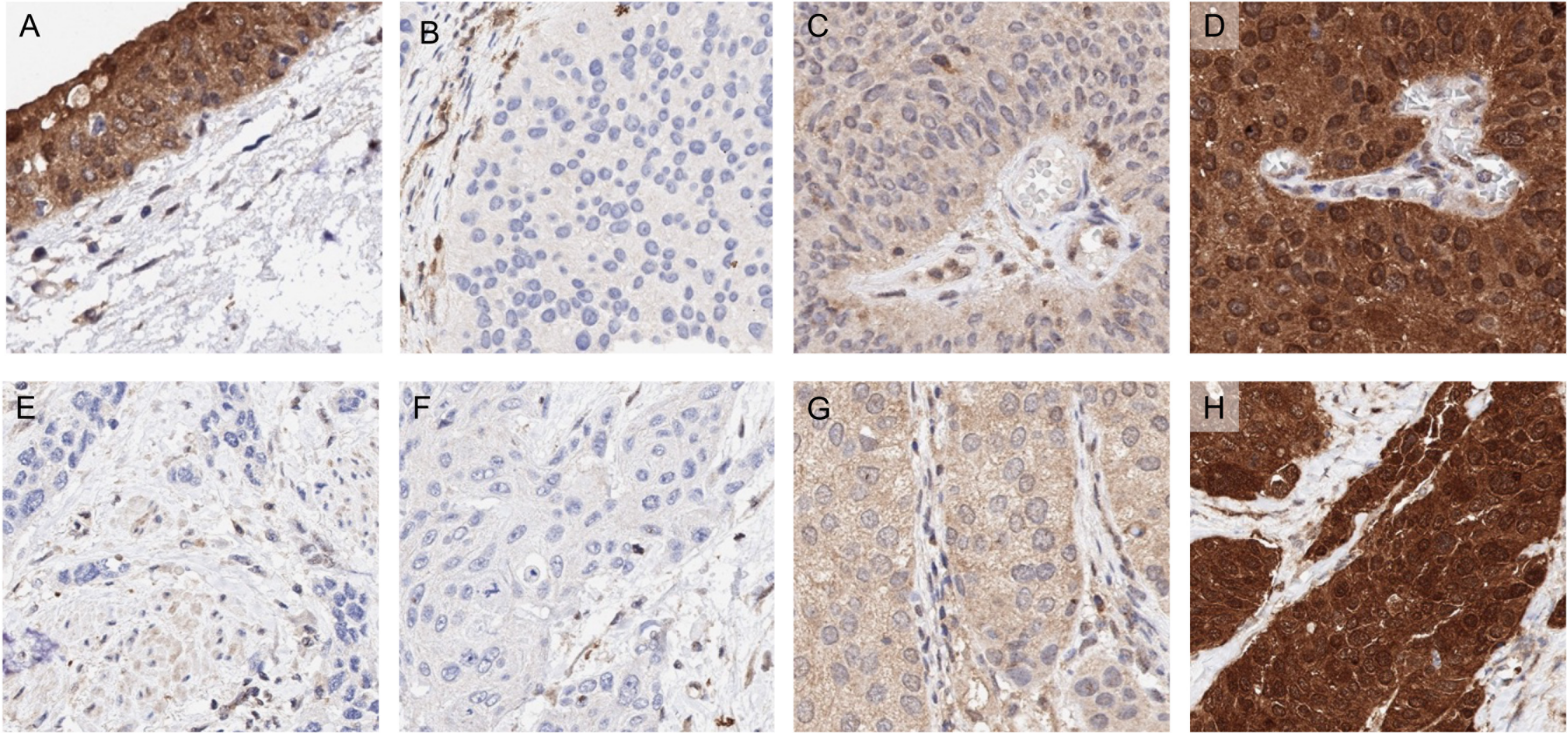
MTAP immunostaining. The panels show (A) strong cytoplasmic and nuclear staining of normal urothelium, (B) absence of MTAP staining in a non‐muscle‐invasive urothelial carcinoma (pTaG2, low‐grade) with homozygous 9p21 deletion, (C) low‐level MTAP staining in a pTaG2 (low‐grade) tumor with a heterozygous 9p21 deletion, (D) strong MTAP staining in a pTaG2 (low‐grade) tumor without 9p21 deletion, (E and F) complete lack of MTAP staining in two cases of muscle‐invasive urothelial carcinoma (pT2–4) with homozygous 9p21 deletion, (G) low MTAP staining in a pT2–4 carcinoma with heterozygous 9p21 deletion, and (H) strong MTAP staining in a pT2–4 carcinoma without 9p21 deletion. All samples contained stroma or muscle cells showing unequivocal MTAP staining.

**Figure 2:**
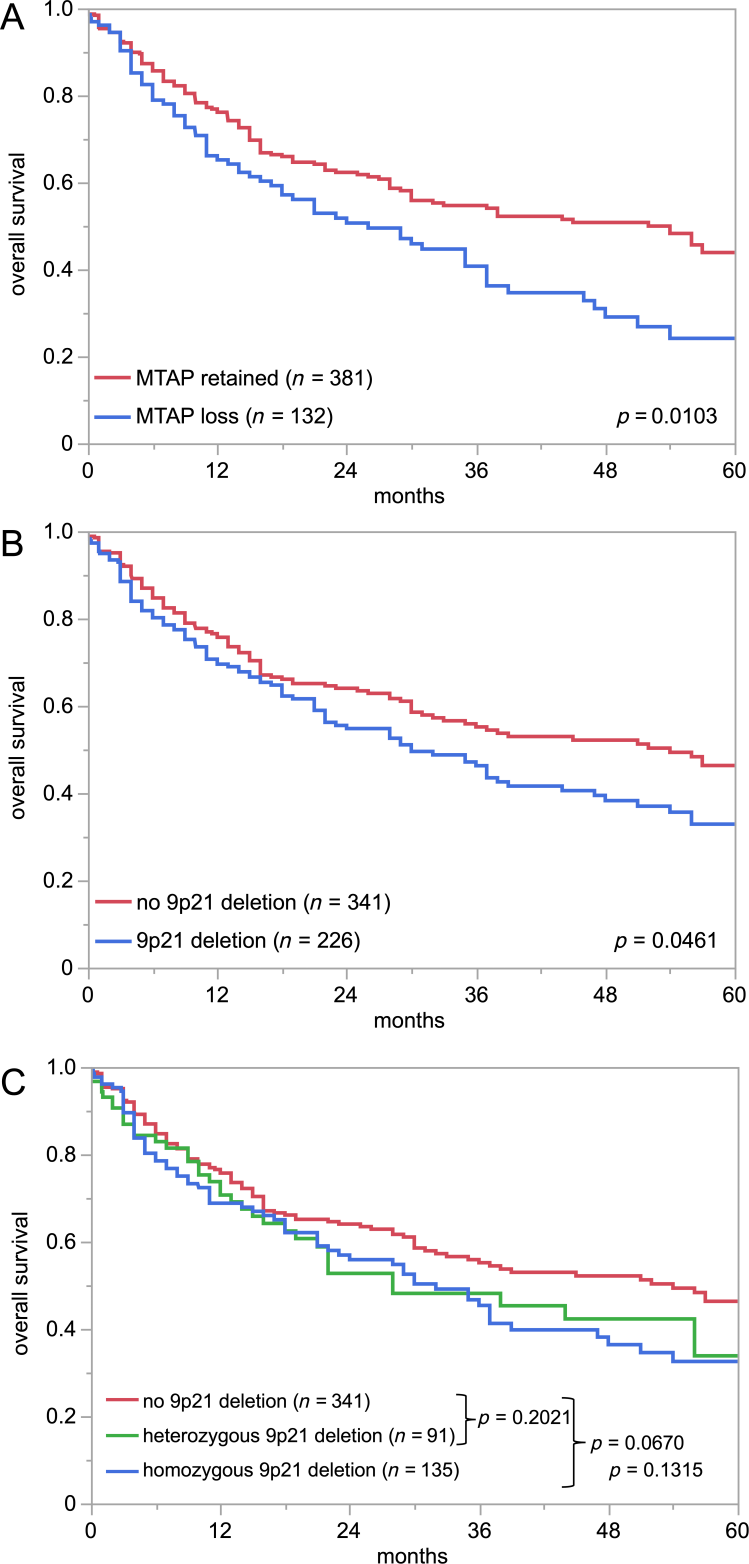
MTAP immunostaining and 9p21 copy number status versus patient prognosis. (A) MTAP immunostaining and overall survival, (B) 9p21 copy number status (nondeleted versus deleted) and overall survival, and (C) 9p21 copy number status (nondeleted versus heterozygous and homozygous deletion) and overall survival. The *p* values in the lower right corner are overall *p* values. The *p* values after the brackets refer to individual groups.

Cytoplasmic and nuclear MTAP immunostaining was abundant in normal urothelium. In urothelial carcinoma, MTAP expression was considered strong in 12.8%, moderate in 32.9%, and weak in 30.3% while it was completely absent in 24.0% of cases. Representative images are shown in Figure 1. Analysis of the whole sections of 10 tumors revealed completely concordant (homogenous positive or negative) immunostaining results as compared to the results of the corresponding TMA spots. The relationship between MTAP staining and tumor phenotype is shown in Table 2. In noninvasive urothelial carcinomas, MTAP expression loss was more common in pTaG2 high‐grade (30.7%) than in pTaG2 low‐grade (10.8%) or pTaG3 carcinomas (17.9%; *p* < 0.0001). MTAP expression loss was more common in muscle‐invasive urothelial carcinomas (pT2–4, 26.8%) than in pTaG3 carcinomas (17.9%; *p* = 0.0345). Among the muscle‐invasive subset, the fraction of tumors with complete MTAP expression loss increased from pT2 (22.2%) to pT3 (30.0%) and pT4 (31.2%; *p* = 0.0193) but it was statistically unrelated to grade, nodal status, L‐ and V‐status. The OS of patients with complete MTAP expression loss in pT2–4 cancers was worse than for patients with retained MTAP expression (*p* = 0.0103; Figure 2A). This also held true in the subset of 317 patients with MTAP IHC data who had never been treated with CPIs (*p* = 0.0018; supplementary material, Figure S1A).

**Table 2:** MTAP immunostaining and 9p21 copy number status versus tumor phenotype

	MTAP immunostaining						9p21 copy number status				
	*n*	0 (%)	1+ (%)	2+ (%)	3+ (%)	*p*	*n*	Homozygous deletion (%)	Heterozygous deletion (%)	No deletion (%)	*p*
All tumors	1792	24.0	30.3	32.9	12.8		1711	21.3	19.5	59.2	
pTaG2 low	305	10.8	20.3	41.3	27.5	<0.0001*	314	9.2	25.8	65.0	<0.0001*
pTaG2 high	127	30.7	18.1	37.8	13.4		129	32.6	20.9	46.5	
pTaG3	78	17.9	26.9	44.9	10.3		36	16.7	16.7	66.7	
pT2	320	22.2	33.4	33.1	11.3	0.0193^†^	297	16.5	18.9	64.6	0.0014^†^
pT3	470	30.0	36.6	27.2	6.2		429	28.7	18.2	53.1	
pT4	234	31.2	35.0	26.9	6.8		203	28.6	15.8	55.7	
Missing data	258						303				
G2	75	37.3	37.3	20.0	5.3	0.1313^†^	50	34.0	24.0	42.0	0.0831^†^
G3	927	27.3	35.0	29.8	8.0		858	24.4	17.5	58.2	
Missing data	22						21				
pN0	515	28.2	36.3	27.6	8.0	0.9462^†^	477	24.5	18.4	57.0	0.6287^†^
pN+	352	28.1	34.7	29.3	8.0		312	27.6	17.3	55.1	
Missing data	157						140				
R0	444	25.9	35.6	28.6	9.9	0.2209^†^	398	23.9	19.1	57.0	0.2720^†^
R1	114	33.3	27.2	31.6	7.9		96	31.3	14.6	54.2	
Missing data	466						435				
L0	198	30.8	33.8	28.3	7.1	0.2171^†^	167	25.7	25.1	49.1	0.0412^†^
L1	228	24.1	32.5	32.0	11.4		203	25.1	15.3	59.6	
Missing data	598						559				
V0	344	25.3	34.9	30.2	9.6	0.2645^†^	288	22.9	21.9	55.2	0.0390^†^
V1	127	34.6	30.7	26.8	7.9		117	34.2	14.5	51.3	
Missing data	553						524				

G, grade; L, lymphatic invasion; pN, pathological lymph node status; pT, pathological tumor stage; R, resection margin status; V, venous invasion.

* Only in pTa carcinomas.

^†^ Only in pT2–4 carcinomas.

### 9p21 FISH

**Figure 3:**
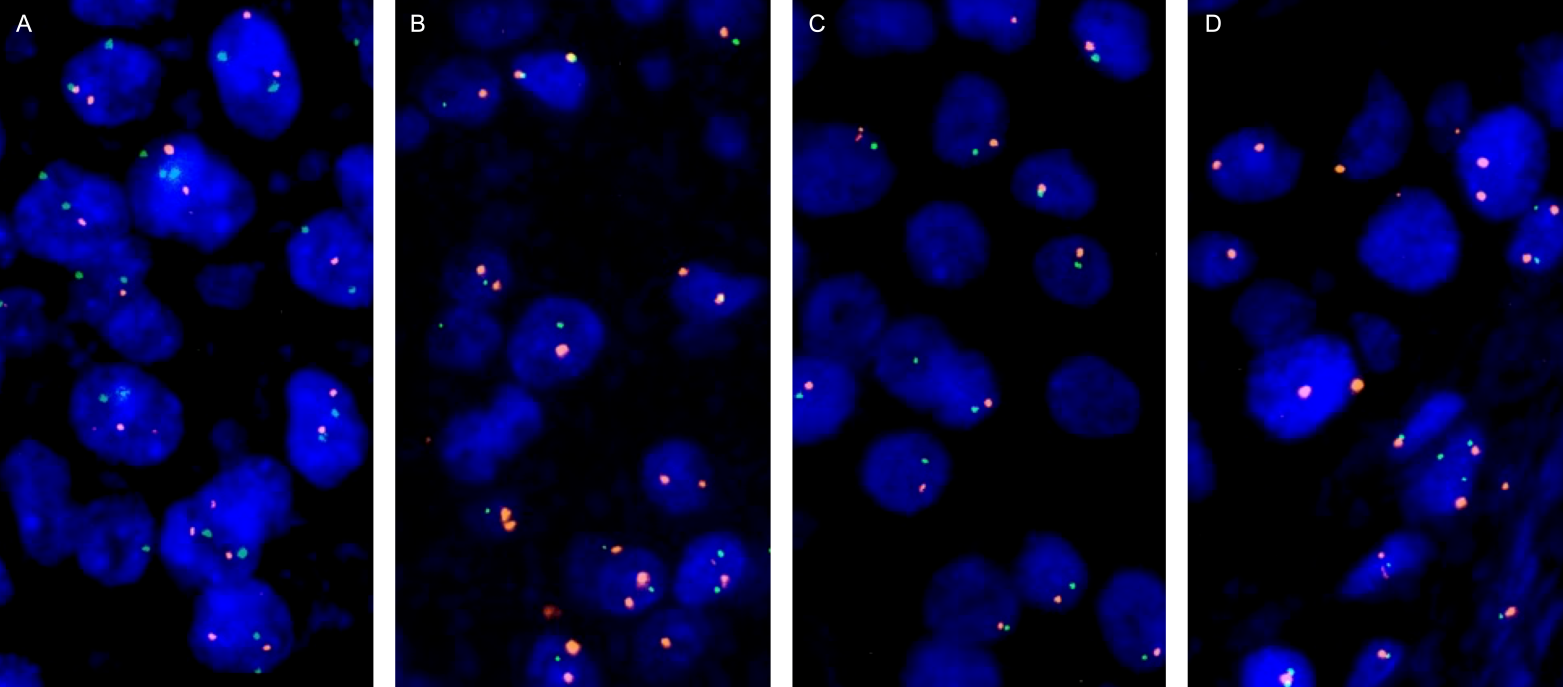
Examples of 9p21 copy number status. (A) No deletion with two green 9p21 signals and two orange centromere 9 signals in tumor cell nuclei, (B) heterozygous 9p21 deletion with one green 9p21 signal and two orange centromere 9 signals in tumor cell nuclei, (C) chromosome 9 monosomy with one green 9p21 and one centromere 9 signal in tumor cell nuclei, and (D) homozygous 9p21 deletion with no green 9p21 signal in all tumor cell nuclei but two green 9p21 signals in adjacent normal cell nuclei and two orange centromere 9 signals in tumor and normal cell nuclei.

**Figure 4:**
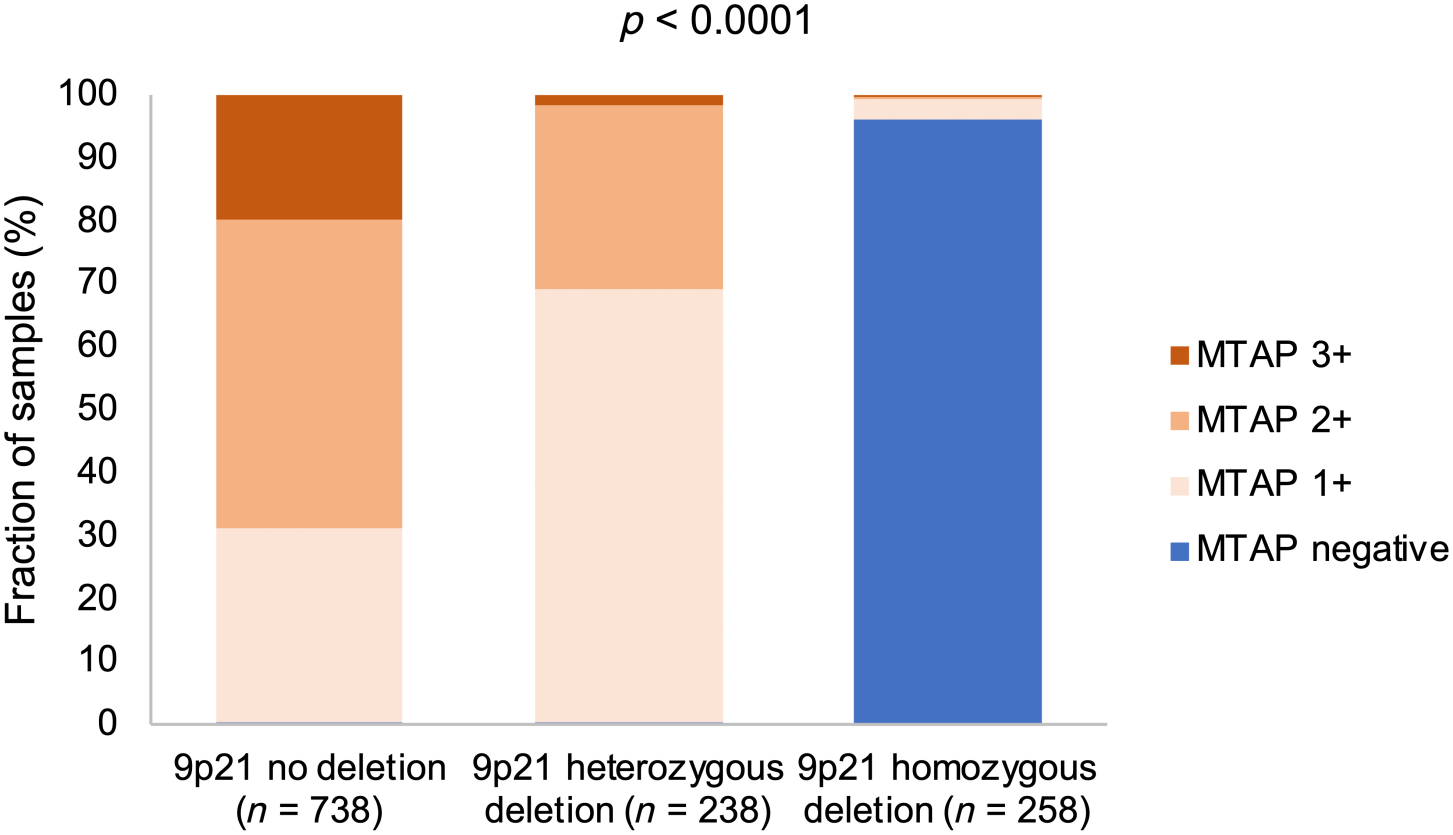
MTAP immunostaining and 9p21 copy number status in urothelial bladder carcinomas. Shown is the intensity of MTAP immunostaining (0, 1+, 2+, 3+) compared to the 9p21 copy number status (no deletion, heterozygous deletion, homozygous deletion). The *p* value refers to the comparison of all groups.

Of the 1,711 informative cases, 21.3% had homozygous 9p21 deletion, 13.7% heterozygous 9p21 deletion, 5.8% monosomy 9 (equivalent to heterozygous 9p21 deletion), and 59.2% had a normal 9p21 status. Representative images are shown in Figure 3. There was a near‐complete concordance between MTAP immunostaining and 9p21 copy number status (Figure 4). Of the 252 tumors with complete MTAP expression loss, 98.4% had homozygous 9p21 deletion while only 4 of 252 MTAP‐negative cases (1.6%) had retained at least one 9p21 copy (*p* < 0.0001). Moreover, 68.9% of the 238 tumors with a heterozygous 9p21 deletion showed weak (reduced; 1+) MTAP immunostaining while weak MTAP staining was only seen in 30.9% of 738 tumors without 9p21 deletion (*p* < 0.0001). The relationship between 9p21 deletion and tumor phenotype was similar to that seen for MTAP IHC (Table 2). In noninvasive urothelial carcinomas, especially, homozygous 9p21 deletion was more common in pTaG2 high‐grade (32.6% homozygous; 20.9% heterozygous) than in pTaG2 low‐grade (9.2%/25.8%) or pTaG3 carcinoma (16.7% each; *p* < 0.0001). The 9p21 deletion was only slightly more common in muscle‐invasive urothelial carcinoma (pT2–4, 23.3%/17.9%) than in pTaG3 carcinoma (*p* > 0.5 for pTaG3 versus pT2–4). Within muscle‐invasive carcinoma, the fraction of tumors with homozygous 9p21 deletion increased from pT2 (16.5%) to pT3 (28.7%) and pT4 (28.6%; *p* = 0.0014) but it was statistically unrelated to grade, nodal status, and L‐ and V‐status. The 9p21 deletions were associated with poor OS in pT2–4 cancer patients (*p* = 0.0461; Figure 2A; *p* = 0.0182 for the subset of 341 patients who had never been treated with CPIs; supplementary material, Figure S1B), but the difference in survival did not reach statistical significance when cases with homozygous and heterozygous 9p21 deletion were separately assessed (Figure 2C).

### Multivariate analyses

In multivariate analyses including pT and pN, both MTAP loss (*p* = 0.0062) and 9p21 deletions (*p* = 0.0407) were independent predictors of prognosis in pT2–4 cancers (Table 3).

**Table 3:** Multivariate analysis including pT, pN, MTAP immunostaining, or 9p21 copy number status

	Parameter		Risk ratio (95% CI)	*p*	Effect summary *p*
9p21 FISH	Tumor stage	pT4 versus pT3	1.9 (1.3–2.6)	0.0002	<0.0001
		pT3 versus pT2	1.5 (1.1–2.2)	0.0228	
		pT2 versus pT1	1.1 (0.44–2.85)	0.8199	
	Nodal stage	pN+ versus pN0	1.8 (1.3–2.4)		<0.0001
	9p21 FISH	Deletion versus no deletion	1.3 (1.0–1.8)		0.0407
MTAP IHC	Tumor stage	pT4 versus pT3	1.5 (1.1–2.1)	0.0140	<0.0001
		pT3 versus pT2	1.8 (1.2–2.7)	0.0021	
		pT2 versus pT1	1.3 (0.4–4.2)	0.6788	
	Nodal stage	pN+ versus pN0	1.9 (1.4–2.6)		<0.0001
	MTAP IHC	Negative versus positive	1.5 (1.1–2.1)		0.0062

FISH, fluorescence *in situ* hybridization; IHC, immunohistochemistry; pN, pathological lymph node status; pT, pathological tumor stage.

### 9p21/MTAP and tumor microenvironment

The relationship between MTAP expression, 9p21 deletions, and various parameters of the tumor microenvironment in muscle‐invasive cancers is shown in Table 4. The 9p21 deletions were associated with low numbers of CD8‐positive (*p* = 0.0103) and CD4‐positive (*p* = 0.0089) lymphocytes, M2 macrophages (*p* < 0.0001), and dendritic cells (*p* = 0.0007). A similar tendency was observed for loss of MTAP expression but statistical significance was reached only for M2 macrophages (*p* = 0.0010) and dendritic cells (*p* = 0.0116). Tumors with 9p21 deletions and loss of MTAP expression were further characterized by low numbers of PD‐L1‐positive tumor cells (*p* < 0.0001 each) and macrophages (*p* = 0.0020 for MTA IHC; *p* < 0.0001 for 9p21 FISH; Table 5).

**Table 4:** MTAP immunostaining and 9p21 copy number status and parameters of the tumor microenvironment in pT2–4 bladder cancers

Cell type	MTAP IHC	Cell density, mean ± SE	*p*	Cell type	9p21 FISH	Cell density, mean ± SE	*p*
CD8 T cells	3+ (*n* = 116)	82.9 ± 13.5	0.0536	CD8 T cells	No deletion (*n* = 703)	93.7 ± 5.6	0.0103
	2+ (*n* = 360)	95.9 ± 7.7			Heterozygous deletion (*n* = 218)	68.5 ± 10	
	1+ (*n* = 406)	69.5 ± 7.2			Homozygous deletion (*n* = 280)	66.7 ± 8.8	
	0 (*n* = 321)	70.9 ± 8.1					
CD4 T cells	3+ (*n* = 116)	92.1 ± 22.1	0.2186	CD4 T cells	No deletion (*n* = 703)	165.1 ± 10	0.0089
	2+ (*n* = 360)	135.1 ± 12.5			Heterozygous deletion (*n* = 218)	120.7 ± 18	
	1+ (*n* = 406)	142.4 ± 11.8			Homozygous deletion (*n* = 280)	114.9 ± 15.8	
	0 (*n* = 321)	123.7 ± 13.3					
M1 macrophages	3+ (*n* = 116)	119.9 ± 17.5	0.1458	M1 macrophages	No deletion (*n* = 703)	161.6 ± 7.5	0.2078
	2+ (*n* = 360)	139.1 ± 9.9			Heterozygous deletion (*n* = 218)	151.1 ± 13.6	
	1+ (*n* = 406)	159.6 ± 9.4			Homozygous deletion (*n* = 280)	136.7 ± 12	
	0 (*n* = 321)	136.7 ± 10.5					
M2 macrophages	3+ (*n* = 116)	448.2 ± 48.6	0.0010	M2 macrophages	No deletion (*n* = 703)	530.7 ± 20.2	<0.0001
	2+ (*n* = 360)	514.6 ± 27.6			Heterozygous deletion (*n* = 218)	403 ± 36.2	
	1+ (*n* = 406)	425.8 ± 26			Homozygous deletion (*n* = 280)	332.2 ± 31.9	
	0 (*n* = 321)	352.9 ± 29.2					
Dendritic cells	3+ (*n* = 116)	323.6 ± 41.2	0.0116	Dendritic cells	No deletion (*n* = 703)	417.2 ± 17.6	0.0007
	2+ (*n* = 360)	400.9 ± 23.4			Heterozygous deletion (*n* = 218)	343.5 ± 31.6	
	1+ (*n* = 406)	347.8 ± 22			Homozygous deletion (*n* = 280)	297.5 ± 27.9	
	0 (*n* = 321)	289.2 ± 24.7					

FISH, fluorescence *in situ* hybridization; IHC, immunohistochemistry.

**Table 5:** MTAP immunostaining, 9p21 copy number status, and PD‐L1 immunostaining in pT2–4 bladder cancers

	PD‐L1 in tumor cells (%)				PD‐L1 in immune cells (%)					
	*n*	Negative	Positive	*p*	*n*	Very few	Few	Some	Many	*p*
MTAP IHC 3+	117	63.2	36.8	<0.0001	117	56.4	25.6	13.7	4.3	<0.0001
MTAP IHC 2+	370	63	37		369	43.1	32.5	21.7	2.7	
MTAP IHC 1+	423	73.3	26.7		423	57.4	28.1	11.6	2.8	
MTAP IHC 0	336	79.2	20.8		336	59.8	29.8	9.2	1.2	
9p21 no deletion	719	63.3	36.7	<0.0001	718	47.4	31.3	18.1	3.2	0.0020
9p21 heterozygous deletion	219	71.2	28.8		219	54.8	28.8	14.2	2.3	
9p21 homozygous deletion	286	76.9	23.1		286	60.8	27.6	10.1	1.4	

IHC, immunohistochemistry.

## Discussion

The results of this study identify about 25% of urothelial carcinomas of the urinary bladder as MTAP‐deficient and demonstrate that MTAP deficiency goes along with a noninflamed tumor microenvironment, aggressive tumor phenotype, and poor patient prognosis in muscle‐invasive disease.

The near‐complete concordance between homozygous 9p21 deletions and a complete loss of MTAP immunostaining is a pivotal result of this study. These consistent data were enabled by optimal and highly validated methods. Although most recent studies have used loss of heterozygosity or comparative genomic hybridization for 9p deletion assessment (supplementary material, Table S2 [20, 21, 22, 31, 32, 33, 34, 35, 36, 37, 38, 39, 40, 41, 42, 43, 44, 45, 46, 47, 48, 49, 50, 51, 52, 53, 54, 55, 56, 57, 58, 59, 60, 61, 62, 63, 64, 65, 66, 67, 68, 69, 70, 71, 72, 73, 74, 75, 76, 77, 78, 79, 80, 81, 82, 83]), FISH is the gold standard for the identification of chromosomal deletions in tumors because alterations can be identified on a single‐cell level [84]. Our MTAP antibody has previously been shown to be highly sensitive and specific for MTAP protein in a validation study on 76 different normal tissue categories [85]. Among the tumors that showed complete loss of MTAP expression loss, 98.4% had a homozygous 9p21 deletion while 1.6% of MTAP‐negative tumors had at least one 9p21 copy retained. This demonstrates that MTAP IHC is reliable for the detection of homozygous 9p21 deletions. Although TMAs are suitable for the comparison of technical methods of detection, it is still possible that small cell populations with homozygous 9p21 deleted were not present on the tissue spots evaluated by MTAP IHC in the few discordant samples as these analyses were not done on consecutive tissue sections. While other mechanisms such as promoter methylation may contribute to MTAP expression loss in rare cases, our data demonstrate that biallelic genomic loss is the major (and probably only) mechanism that can cause complete MTAP deficiency in urothelial carcinoma. Besides, heterozygous 9p21 deletions and monosomy 9 were significantly associated with reduced/low MTAP expression. This suggests that reduced, in addition to absent, MTAP immunostaining may also have diagnostic and clinical implications.

Both our FISH and IHC data suggest a role for MTAP deficiency in grade and stage progression of urothelial neoplasms. This is illustrated by the significant increase of homozygous 9p21 deletions and MTAP expression loss from pTaG2 low‐grade to pTaG2 high‐grade and from pTaG3 to pT2 and pT3/4. The worse OS of MTAP‐deficient as compared to MTAP‐proficient tumors is also consistent with a role for MTAP loss in tumor progression. In concordance with these findings, Vlajnic *et al* [24] found a significantly higher frequency of MTAP deficiency in metastases (53%) than in primary tumors from pT2–4 urothelial carcinomas (33%). MTAP deficiency has also been linked to cancer progression in other tumor entities. For example, Su *et al* [86] found that loss of MTAP immunostaining was independently linked to poor patient prognosis in non‐small cell lung cancers while Abrahao‐Machado *et al* [87] described a significant association of MTAP staining loss with poor patient outcome in Ewing family sarcomas.

In view of the proposed role of MTAP deficiency as a predictor of poor response to CPIs, we made use of the tumor microenvironment data from earlier studies to evaluate the possible interactions of MTAP deficiency with antitumor immunity [29]. The significantly lower rate of PD‐L1‐positive tumor cells and macrophages, as well as of intratumoral CD8‐positive and CD4‐positive lymphocytes, M2 macrophages, and dendritic cells in MTAP‐deficient urothelial carcinomas is consistent with more efficient immune evasion in these tumors. Because the *type‐I interferon* gene cluster on 9p21.3 is often involved in 9p21 deletions, which also involve MTAP, has been suggested that this deletion may result in reduced expression of a number of genes – for example, *CXCL13*, *CXCL9*, *XCL2*, *CD27*, and *IL21* – by tumor cells and subsequently disable both cell‐intrinsic and cell‐extrinsic tumor suppression, facilitating tumor cells to escape from CD8 T‐cell surveillance [16]. In line with this notion, recent studies have described a worse response of 9p21/MTAP‐deficient melanoma, non‐small cell lung cancer, and metastatic urothelial cancer to CPIs as compared to 9p21/MTAP‐proficient cancers [16, 88, 89]. It appears possible that the observed link between MTAP deficiency and unfavorable tumor phenotype is driven by the markedly lower level of measurable immune response in the tumor microenvironment of MTAP‐deficient cancers. In urothelial carcinoma, the quantity of intratumoral immune cells has a pivotal impact on patient prognosis [29].

Given that MTAP expression was always strong in normal (non‐neoplastic) urothelium, and that complete MTAP expression loss was strictly limited to subsets of urothelial neoplasms, complete loss of MTAP expression can be considered a reliable marker for neoplastic urothelium. MTAP IHC may thus become a highly useful tool for the distinction of neoplastic from non‐neoplastic urothelium. The distinction of neoplastic from non‐neoplastic urothelium cannot reliably be made by morphology alone if low‐grade urothelial neoplasia occurs in flat urothelium (dysplasia versus reactive changes), cytologic specimens, or in very small and crushed biopsies [90, 91]. Although not all patients with urothelial neoplasia can benefit from MTAP IHC, this approach appears to be highly useful in subsequent biopsies or cytological samples of patients with a previously diagnosed MTAP‐deficient urothelial neoplasm. In these patients, absent or markedly reduced MTAP staining may indicate flat dysplasia. In a recent study from Vlajnic *et al* [24], MTAP IHC was applied to 729 consecutive urothelial carcinomas from routine practice. In line with our data, these authors found MTAP deficiency in 22% of their patients, identified tumor‐adjacent dysplastic urothelium solely on the basis of unequivocal MTAP negativity in several cases, and found persistent MTAP deficiency in 37 of 38 subsequent recurrences [24].

In summary, the results of our study show that complete MTAP expression loss occurs in 20–30% of urothelial carcinomas and that MTAP expression loss is restricted to neoplastic urothelium carrying homozygous 9p21 (MTAP) deletions. Accordingly, MTAP expression loss is a marker for neoplastic urothelium. Based on the results of this study and the data from Vlajnic *et al* [24], we are now routinely applying MTAP IHC to every newly diagnosed urothelial carcinoma and in cases of flat urothelium with atypia of unknown significance. This procedure at least enables us to identify these patients for which MTAP IHC will be potentially instrumental in subsequent biopsies or cytological specimens. Moreover, MTAP deficiency appears to identify tumors with a critical vulnerability to drugs targeting *de novo* adenine biosynthesis and (possibly) increased resistance toward CPIs.

## Author contributions statement

NG, NW, MK, RS and GS contributed to the conception, design, data collection, data analysis and manuscript writing. NG, NW, HP, ML, NCB, AHM, HS, SS, FR, SE, SK, SM and DH participated in pathology data analysis and data interpretation. VA and MK carried out FISH analysis. NG, HP, SH, KF, SW, PGB, SS, FR, SE, ML, NCB, AHM, HS, MF, MR, MS, KK, TE, TK, SK, NA, SM, HS, HZ, DH, TS and LB collected samples. RS and MK carried out data analysis. NG, MK, GS, RS and TS supervised the study. All authors agree to be accountable for the content of the work.

## Supporting information


**Figure S1.** Prognostic impact of MTAP IHC and 9p21 deletion status in patients with muscle‐invasive urinary bladder cancer who were treated by radical cystectomy before 2017 when immune checkpoint therapies became available
**Table S1.** List of the antibodies, antigen retrieval and dilutions used for multiplex fluorescence immunohistochemistry in the work of Debatin *et al* [29]
**Table S2.** Summary of previous 9p21 copy number studies

## Data Availability

Raw data are available upon reasonable request. All data relevant to the study are included in the article.
