# Supplementary material for: Loss of MTAP expression is strongly linked to homozygous 9p21 deletion, unfavorable tumor phenotype, and noninflamed microenvironment in urothelial bladder cancer
Source: J Pathol Clin Res. 2024 Dec 12;11(1):e70012. doi: 10.1002/2056-4538.70012 (PMC11638363; doi:10.1002/2056-4538.70012)
Supplement: Supplementary file 1 — Figure S1. Prognostic impact of MTAP IHC and 9p21 deletion status in patients with muscle‐invasive urinary bladder cancer who were treated by radical cystectomy before 2017 when immune checkpoint therapies became available Table S1. List of the antibodies, antigen retrieval and dilutions used for multiplex fluorescence immunohistochemistry in the work of Debatin et al [29] Table S2. Summary of previous 9p21 copy number studies [file CJP2-11-e70012-s001.pdf]

Loss of MTAP expression is strongly linked to homozygous 9p21 deletion, unfavorable tumor phenotype, and noninflamed microenvironment in urothelial bladder cancer

N Gorbokon *et al*. *J Pathol Clin Res* https://doi.org/10.1002/2056-4538.70012

Supplementary Figure S1
Supplementary Tables S1 and S2

Reference numbers refer to the list in the main paper

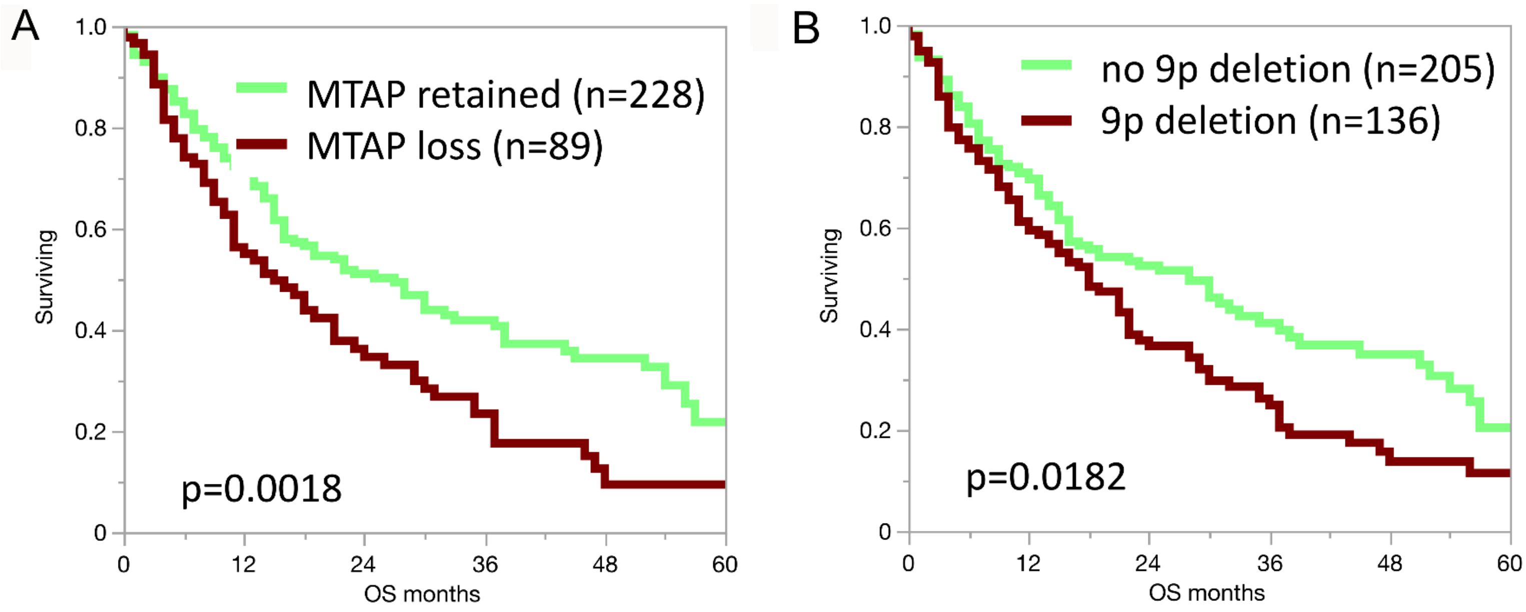

Figure S1. Prognostic impact of (A) MTAP IHC and (B) 9p21 deletion status in patients with muscle-invasive urinary bladder cancer who were treated by radical cystectomy before 2017 when immune checkpoint therapies became available

Table S1. List of the used antibodies, antigen retrieval, and dilutions, used for multiplex fluorescence immunohistochemistry in the work of Debatin *et al* [29]

Antibody name	Antibody vendor and clone	Antigen retrieval (pH)	Dilution
CD68	Agilent/DAKO, Clone: PG-M1<br>Cat#: IR613	9.0	1:3
TIM-3	MSVA, Clone: MSVA-366R<br>Cat#: 3484-366R-01	9.0	1:150
PD-L1	MSVA, Clone: MSVA-711R<br>Cat#: 2083-711R	9.0	1:150
CTLA-4	MSVA, Clone: MSVA-152R<br>Cat#: 3451-152R-01	9.0	1:150
PD-1	abcam, Clone: EPR4877(2)<br>Cat#: ab137132	9.0	1:150
CD163	Leica, Clone: 10D6<br>Cat#: NCL-L-CD163	9.0	1:100
CD20	MSVA, Clone: MSVA-020R<br>Cat#: 2483-020R-01	9.0	1:150
CD3	Agilent/DAKO, Clone: Polyclonal<br>Cat#: IR503	9.0	1:3
CD11c	abcam, Clone: EP1347Y<br>Cat#: ab52632	9.0	1:450
FOXP3	BioLegend, Clone: 206D<br>Cat#: 320107	9.0	1:25
CD4	MSVA, Clone: MSVA-004R<br>Cat#: 2278-004R-01	9.0	1:150
CD8	Agilent/DAKO, Clone: C8/144B<br>Cat#: IR623	9.0	RTU
panCK	MSVA, Clone: MSVA-000R<br>Cat#: 2105-000R-01	9.0	1:150
Ki-67	MSVA, Clone: MSVA-267M<br>Cat#: 2082-267M-01	9.0	1:150
CD31	MSVA, Clone: MSVA-031M<br>Cat#: 2517-031M-01	9.0	1:150
Vimentin	Agilent/DAKO, Clone: V9<br>Cat#: IR630	9.0	RTU

HLA-DRa	MSVA, Clone: MSVA-470R<br>Cat#: 3328-470R-01	9.0	1:150
Myosin-11	MSVA, Clone: MSVA-375R<br>Cat#: 2450-375R	9.0	1:150
Desmoglein 3	MSVA, Clone: MSVA-543M<br>Cat#: 4355-543M-01	9.0	1:150
PAX-8	GeneAB, Clone: IHC008<br>Cat#: IHC008-1	9.0	1:30
CDH16	MSVA, Clone: MSVA-516R<br>Cat#: 4036-516R-01	9.0	1:150

Table S2. Summary of previous 9p21 copy number studies

Author, year	PMID	Method	Analyzed tumors	9p21 deletion in total (%)	Heterozygous deletion (%)	Homozygous deletion (%)	Monosomy 9 (%)
Perrino *et al*, 2019 [31]	31054897	FISH	15	60%	x	x	x
Park *et al*, 1996 [32]	8843005	LOH	28	61%	36%	25%	x
Keck *et al*, 2011 [33]	20878954	FISH	31	85%	x	15%	x
Williamson *et al*, 1995 [34]	8541841	LOH	140	39%	12%	27%	x
Orlow *et al*, 1995 [35]	7563186	Southern blot	100	19%	8%	15%	x
Hu *et al*, 2020 [36]	32974362	FISH	5	20%	x	x	x
Böhm *et al*, 1997 [37]	9221807	LOH	84	33%	x	33%	x
Stadler *et al*, 2001 [38]	11410506	FISH	55	67%	x	31%	5%
Hartmann *et al*, 2000 [39]	10830781	FISH	52	90%	x	31%	60%
Chang *et al*, 2003 [40]	12853838	Southern blot	53	43%	x	43%	x
Riesz *et al*, 2007 [41]	17922047	FISH	34	32%	x	x	x
Baud *et al*, 1998 [42]	9714047	LOH	44	96%	x	x	32%
Kim *et al*, 2019 [43]	31289598	FISH	27	19%	x	18.5%	x
Hartmann *et al*, 1999 [44]	10079249	FISH	14	50%	29%	14%	29%
Yurakh *et al*, 2006 [45]	16624482	LOH	84	24%	x	20%	x
Berggren de Verdier *et al*, 2006 [46]	17060081	qPCR	478	15%	x	15%	x
Tsutsumi *et al*, 1998 [47]	9881704	LOH	31	45%	x	45%	x
Southgate *et al*, 1995 [48]	7577470	PCR	13	54%	x	54%	x
Hopman *et al*, 2002 [49]	12368185	FISH	22	18%	x	18%	x
Obermann *et al*, 2004 [50]	15010867	FISH	89	16%	x	x	x
Frère-Belda *et al*, 2001 [51]	11720438	PCR	28	18%	x	18%	x
Balázs *et al*, 1997 [52]	9171998	FISH	17	35%	x	35%	x
Florl *et al*, 2000 [53]	11045568	LOH	86	40%	x	20%	x
Zellweger *et al*, 2006 [54]	16646074	FISH	138	15%	x	15%	x

Yin *et al*, 2008 [55]	18234280	FISH	31	42%	x	x	x
Hafner *et al*, 2001 [56]	11521204	LOH	94	46%	46%	x	x
Sassa *et al*, 2019 [57]	30668617	FISH	39	28%	x	x	x
Rosin *et al*, 1995 [58]	7585577	LOH	31	61%	x	29%	x
Prat *et al*, 2001 [59]	11337313	CGH	24	25%	x	x	x
Heidenblad *et al*, 2008 [60]	18237450	aCGH	38	32%	x	32%	x
Gallucci *et al*, 2007 [22]	17950275	FISH	62	79%	31%	48%	x
Sauter *et al*, 1995 [61]	7615360	FISH	162	29%	x	x	29%
Cairns *et al*, 1995 [62]	7550353	PCR+LOH	285	44%	x	44%	x
Schwarz *et al*, 2008 [63]	17693577	FISH	82	54%	x	x	x
Orlow *et al*, 1999 [64]	10393843	PCR	121	14%	3%	11%	x
Packenham *et al*, 1995 [65]	7576106	LOH	28	39%	x	29%	x
Baud *et al*, 1999 [66]	10024675	LOH	44	48%	x	x	x
Berggren *et al*, 2003 [67]	12538475	PCR	186	26%	12%	14%	x
Benedict *et al*, 1999 [20]	10022125	LOH	22	64%	59%	5%	x
Toncheva et Zaharieva, 2005 [68]	15897688	FISH	25	60%	x	x	x
Lin *et al*, 1995 [69]	21224098	LOH	26	27%	x	x	x
Veltman *et al*, 2003 [70]	12782593	PCR	41	34%	x	34%	x
Cairns *et al*, 1998 [71]	9516934	PCR+LOH	59	66%	29%	37%	x
Cheng *et al*, 2005 [72]	15855652	LOH	19	47%	x	x	x
Krüger *et al*, 2003 [21]	12792774	FISH	71	83%	x	83%	x
Amira *et al*, 2003 [73]	14501713	LOH	24	58%	58%	x	x
Jones *et al*, 2005 [74]	16166427	LOH	58	33%	33%	x	x
Jones *et al*, 2005 [75]	16196038	LOH	23	39%	39%	x	x
Bollmann *et al*, 2005 [76]	15892805	FISH	34	50%	x	x	x
Wada *et al*, 2003 [77]	12745717	LOH	124	33%	33%	x	x
Williamson *et al*, 2014 [78]	24743222	FISH	17	18%	x	x	x
Uchida *et al*, 2000 [79]	11127706	LOH	45	47%	x	x	x
Traczyk *et al*, 2011 [80]	24578886	LOH	120	34%	34%	x	x

Lopez-Beltran *et al*, 2006 [81]	16482499	LOH	125	57%	57%	x	x
Ke *et al*, 2014 [82]	24396440	FISH	79	57%	x	51%	x
Bartoletti *et al*, 2007 [83]	17612565	LOH	56	77%	77%	x	x
